# Interpreting *in vivo* calcium signals from neuronal cell bodies, axons, and dendrites: a review

**DOI:** 10.1117/1.NPh.7.1.011402

**Published:** 2019-07-30

**Authors:** Farhan Ali, Alex C. Kwan

**Affiliations:** a Yale University, Department of Psychiatry, School of Medicine, New Haven, Connecticut, United States; b Yale University, Department of Neuroscience, School of Medicine, New Haven, Connecticut, United States

**Keywords:** calcium imaging, two-photon microscopy, fluorescence, neuron, calibration

## Abstract

Calcium imaging is emerging as a popular technique in neuroscience. A major reason is that intracellular calcium transients are reflections of electrical events in neurons. For example, calcium influx in the soma and axonal boutons accompanies spiking activity, whereas elevations in dendrites and dendritic spines are associated with synaptic inputs and local regenerative events. However, calcium transients have complex spatiotemporal dynamics, and since most optical methods visualize only one of the somatic, axonal, and dendritic compartments, a straightforward inference of the underlying electrical event is typically challenging. We highlight experiments that have directly calibrated *in vivo* calcium signals recorded using fluorescent indicators against electrophysiological events. We address commonly asked questions such as: Can calcium imaging be used to characterize neurons with high firing rates? Can the fluorescent signal report a decrease in spiking activity? What is the evidence that calcium transients in subcellular compartments correspond to distinct presynaptic axonal and postsynaptic dendritic events? By reviewing the empirical evidence and limitations, we suggest that, despite some caveats, calcium imaging is a versatile method to characterize a variety of neuronal events *in vivo*.

## 1 Introduction

Calcium imaging refers to optical methods for measuring the concentration of calcium ions in cells. In neuroscience, there has been an explosion in the number of studies that employ calcium imaging. For example, the method has been applied to awake animals to study, at the subcellular level, the role of dendritic calcium signals during perceptual performance;^1^ at the single-neuron level, learning-related neural activity across the course of motor skill acquisition;^2^ at the local-circuit level, dynamics of frontal cortical ensembles during decision-making;^3^ and, at the network level, interactions between brain regions that underlie neurodevelopment.^4^ Calcium imaging is also the primary method behind a recent large-scale effort to survey neural responses in the mouse visual cortex.^5^

The rising popularity of calcium imaging is due in part to technical advances. The development of genetically encoded calcium indicators (GECIs)—fluorescent proteins that sense calcium and report via a change in emission amplitude or spectrum—enabled measurements with high signal-to-noise ratios.^6^ Further work in protein engineering led to GECI variants with attractive properties such as a redshifted emission spectrum.^7^ These GECIs can be introduced not only using viruses but are also available in a variety of transgenic animals.^8^ Moreover, there are mature optical techniques for subcellular-resolution imaging in the intact brain based on two-photon-excited fluorescence.^9^ New microscope designs additionally enable imaging deep in scattering tissues^10^ and across a wide field of view.^11^ Collectively, these advances have led to the widespread adoption of calcium imaging as a tool for neuroscience research.

A major reason for measuring calcium is that the influx of calcium ions is associated with electrical events, such as synaptic activation and dendritic spikes, that are difficult to measure with other methods. The inference is possible because we have accrued knowledge of the biophysical mechanisms that regulate the entry and life cycle of calcium ions in neuronal compartments. The inference is made stronger by computational models that describe calcium measurements in cellular compartments, which allows for a better dissociation between the desired goal to measure free calcium ions and the side effect of buffering by the calcium indicators.^12^^,^^13^ These topics have been discussed extensively in other review articles that have focused on the utility of calcium imaging as a tool in neuroscience,^14^^–^^16^ the mechanisms that regulate calcium in neurons,^17^^–^^20^ the quantitative aspects of sensing calcium with fluorophores,^21^^–^^23^ and procedures for analyzing calcium imaging data.^24^

This article is intended to be a primer for interpreting calcium signals recorded from neurons with fluorescent indicators *in vivo*. Namely, if we see a fluorescent transient in the cell body, axon, or dendrite, what does the signal mean? Are the spatiotemporal dynamics consistent with a cellular event? Moreover, if the signal seems physiological, what can we say about the electrical event that gives rise to the calcium transient, and what *in vivo* calibration data would support the conclusion? Calibration is particularly relevant because *in vivo* neural dynamics are affected by background activity, neuromodulation, and GABAergic inputs, such that calcium signals could be markedly different in *in vivo* versus *in vitro* conditions. With these questions in mind, the main text is divided into sections based on various neural correlates commonly assigned to *in vivo* calcium signals. Within each section, we briefly introduce the biophysical basis, and then focus on experiments that have directly calibrated *in vivo* calcium signals against electrophysiological events. We highlight empirical details that are important for proper interpretation of calcium imaging data. We end each section by discussing limitations. We primarily use neocortical neurons as examples because many types of *in vivo* calibration data are available.

## 2 Somatic Calcium Signals: as a Proxy for Spiking Activity

Somatic calcium signals have been used to infer spiking activity in neurons. By imaging an ensemble of neurons at single-cell resolution, one could delineate task-related neural activity during perceptual or cognitive behavior,^25^ map the spatial organization of visually evoked neural responses,^26^ and track modifications to neural activity across learning.^27^

An action potential causes calcium influx in the neuronal cell body. For example, in layer 5 pyramidal neurons in the neocortex, calcium enters the soma through voltage-dependent calcium channels, including L-type channels that open in response to a large depolarization (∼20  mV), as well as N-, and P/Q-type channels.^28^ The resting calcium concentration at the soma is ∼50  nM, and the increase due to a single action potential is estimated to be ∼40  nM.^29^ This number may underestimate the actual amplitude, because even a moderate amount of fluorescent indicators would buffer and prolong the life cycle of calcium ions in a cell.^13^^,^^30^ Measurements in the nearby proximal apical dendrite (30 to 50  μm from soma) suggest a rapid rise time (<2  ms to 90% of the peak amplitude; the measurement was limited by the kinetics of fluorescent indicators), and a decay time constant of ∼70  ms.^13^ At the cell body, because of larger cytosolic volume, the decay time constant should be larger than the value at the proximal apical dendrite, and one study estimates a factor of ∼150%.^29^ Therefore, although the action potential is a millisecond-scale event, the somatic calcium elevation has a duration that is longer by about two orders of magnitude.

**Fig. 1:**
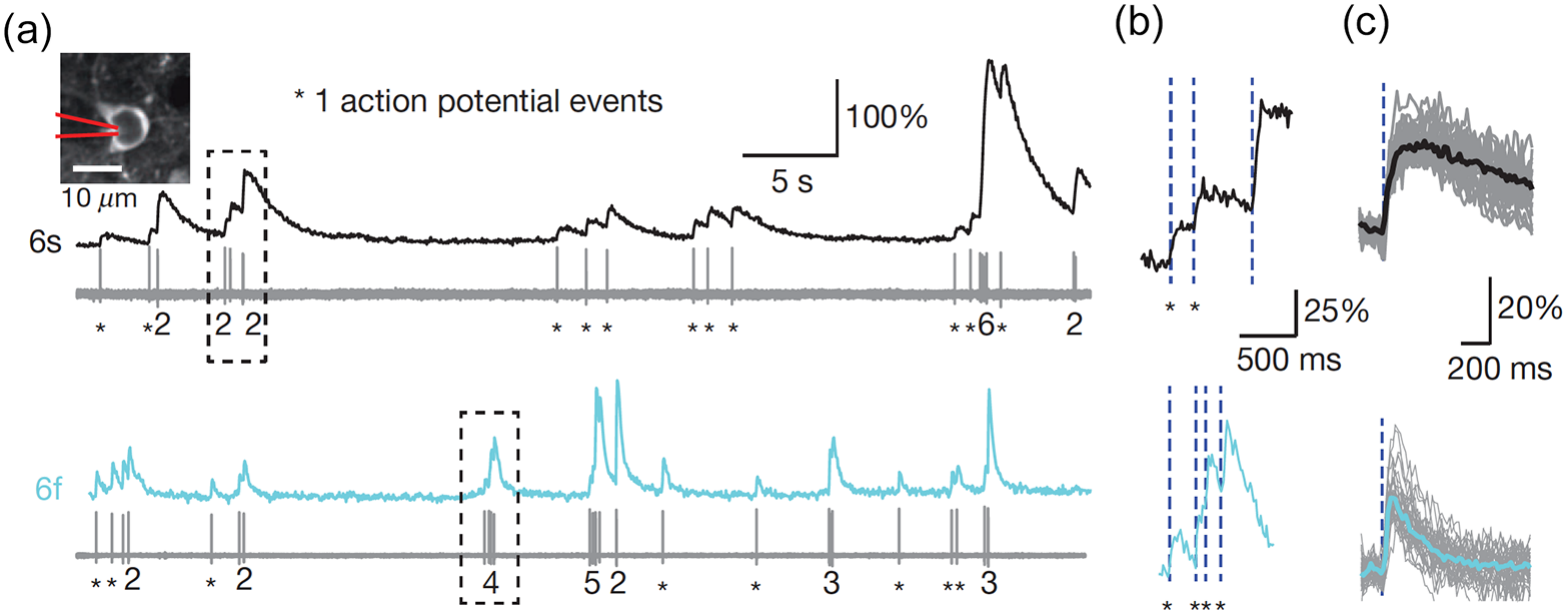
Somatic calcium transients reflect spiking activity of cortical neurons. (a) Somatic calcium transients from a GCaMP6s- (top row) or GCaMP6f-expressing neuron (bottom row) in layer 2/3 of the mouse visual cortex, imaged using a two-photon microscope. Spiking activity was recorded from the imaged neurons using a juxtacellular electrode. The numbers denote the number of action potentials. An asterisk denotes a single spike. (b) Magnified view of the boxes in (a). (c) Median fluorescence transient in response to single action potential events with nearby spikes at least 1 s away. Figure adapted from Ref. 6. Reproduced with permission, courtesy of Springer Nature.

Many studies have imaged calcium signals while recording spiking activity from the cell body *in vivo* to show a direct relationship between somatic calcium transients and action potentials.^6^^,^^31^^–^^33^ In one example,^6^ putative excitatory somata were targeted for simultaneous two-photon calcium imaging using the fluorescent indicator GCaMP6s or GCaMP6f and juxtacellular recording in the neocortex of anesthetized mice [Fig. 1(a)]. Fluorescence rose with each action potential [Fig. 1(b)]. It should be emphasized that the signal reported by a fluorescent indicator is a convolution of the calcium transients and the indicator response. The choice of indicator matters, as evident by the different fluorescence profiles in response to a single spike for the two GECIs [Fig. 1(c)]. In this case, GCaMP6s has slower single-spike kinetics (time to peak of 179 ms and decay time of 550 ms) compared to GCaMP6f (45 and 142 ms, respectively).^6^

Furthermore, in the intervening periods between action potentials, the fluorescence was mostly unchanged [Fig. 1(a)], suggesting that calcium elevations occurred only from suprathreshold depolarizations. This is consistent with the biophysical properties of the high-threshold calcium channels in the cell body. In practice, for cortical pyramidal neurons, it is possible to find a weak correlation between the subthreshold fluctuations in membrane potentials and the somatic calcium signals recorded *in vivo*. However, the source of that weak correlation probably does not come from the imaged cell, but rather originates from contamination of fluorescence signals from the surrounding neuropil, which would reflect the local network activity and correlate with membrane potentials of the imaged cell [for an example, see Supplementary Figs. 1(e) and 1(f) in Ref. 32].

When action potentials are sparse relative to the imaging frame rate, a “kernel” can be estimated to describe the fluorescence response to a single action potential [Fig. 1(c)]. Deconvolution of this kernel from the time-lapse fluorescent signal would yield spike times.^34^^,^^35^ Having the kernel also enables other types of analyses. For example, spike-field coherence has been used to determine phase synchronization between neuronal firing and local oscillatory activity.^36^ Analogously, from calcium imaging and field recordings, a fluorescence-field coherence could be determined. In one study that imaged spinal neurons while recording from motor nerves, the coherence allows for quantification of the locomotion phase during which the neurons contribute to motor pattern generation.^37^ The kernel is required to account for the kinetics of the indicator to ensure the phase is accurate.

In a few cases, such as granule cells in the *Xenopus* tadpole olfactory bulb^38^ and principal neurons in the locust antennal lobe,^39^ the somatic calcium signals do not strictly follow the spiking activity. This suggests that additional sources of calcium, such as low-threshold calcium channels or internal stores, are contributors and cannot be neglected for the soma of those cell types. These counterexamples highlight the need to calibrate for the cell type and animal model of interest.

## 3 Somatic Calcium Signals: for Neurons with High Firing Rates

Some neurons, such as the GABAergic fast-spiking cells in the neocortex, have high firing rates. Although spike inference becomes more difficult, calcium imaging may still be an attractive approach because some interneuron subtypes are few in numbers and sparsely distributed and, therefore, difficult to record with other methods. Applications of interneuron imaging include determining the orientation selectivity of GABAergic neuron subtypes in the rodent visual cortex^40^ and dissecting functional responses of interneuron subtypes during delayed response tasks.^41^

**Fig. 2:**
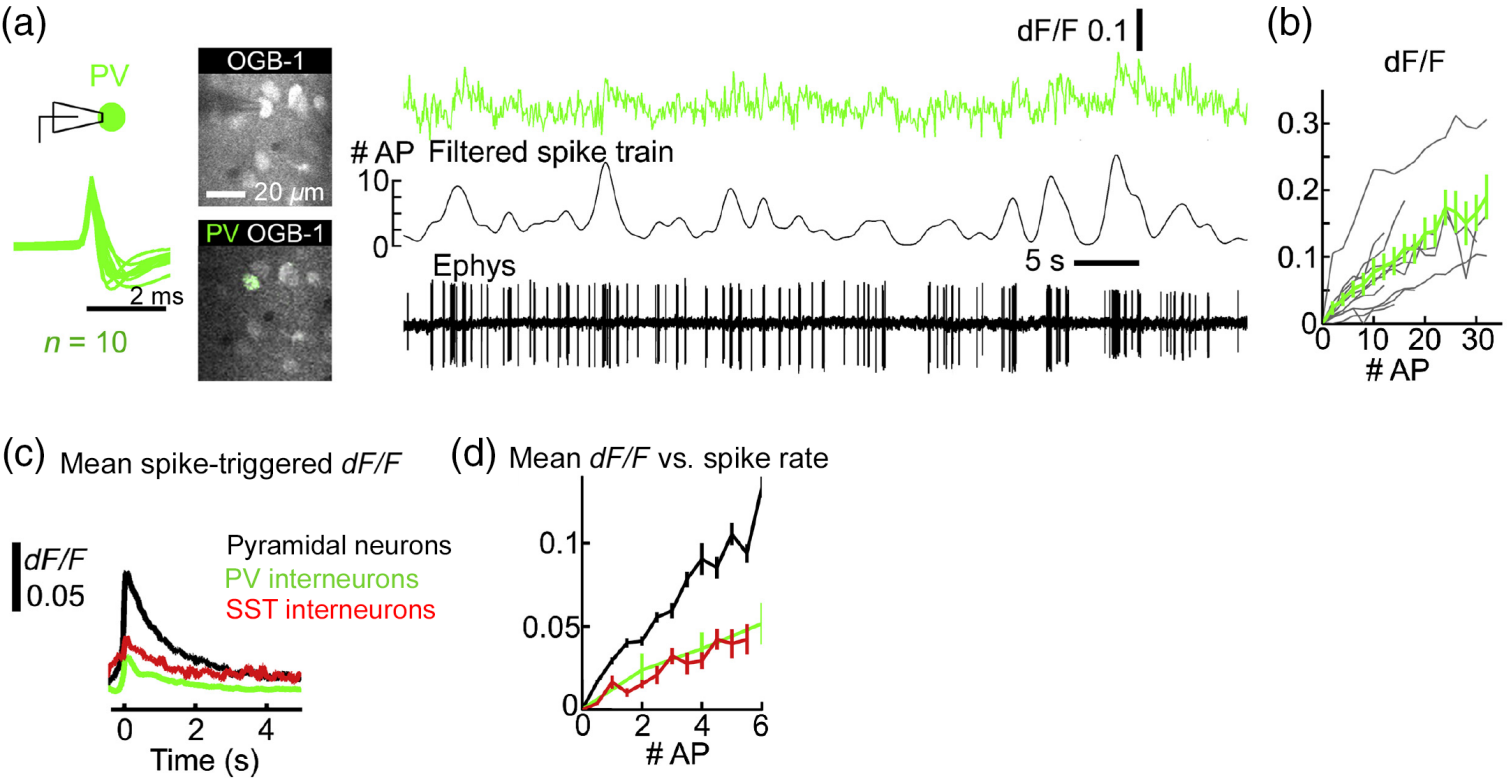
Somatic calcium transients correlate with firing rates of cortical GABAergic neurons. (a) Somatic calcium transients from a parvalbumin-expressing (PV) interneuron in layer 2/3 of the mouse visual cortex, imaged using a two-photon microscope. Cell type was identified based on tdTomato expression. Neurons were loaded with the synthetic calcium dye Oregon Green BAPTA-1 (OGB-1). Spiking activity was recorded under cell-attached condition. The left panel shows the mean normalized spike waveforms of 10 cells. The middle panel shows images including a recorded neuron. The right panel shows fluorescence and electrophysiological traces from an example cell. The filtered spike train was smoothed with a Gaussian filter (s.d. = 0.5 s). (b) Fluorescence versus number of action potentials was determined using the filtered spike train. Gray lines, individual cells. Black line, mean ± s.e.m. (c) Mean spike-triggered fluorescence for excitatory (n=16), PV (n=10), and SST neurons (n=8). (d) Mean fluorescence versus number of action potentials for excitatory, PV, and SST neurons. The figure is adapted from Ref. 32. Reproduced with permission, courtesy of Elsevier.

Several years ago, we performed simultaneous imaging and electrophysiological recording to provide evidence for a direct relationship between somatic calcium signals and firing rates in cortical GABAergic neurons^32^ (Fig. 2). Certain interneurons, such as parvalbumin-expressing (PV) fast-spiking cells, have a high *in vivo* firing rate—up to an instantaneous rate of ∼35  Hz in anesthetized mice in our study and a mean rate of ∼50  Hz in awake, behaving mice.^42^^,^^43^ For several reasons, as detailed at the end of this section, inferring the times of individual action potentials was imprecise in our original study. Nevertheless, most PV cells exhibited fluorescence signals that closely tracked the firing rate [Fig. 2(a)]. Therefore, even in a case when individual spikes could not be resolved, calcium imaging may be used to read out changes in firing rate.

Perhaps the most notable insight from those experiments was the variability. There was variability across cells belonging to the same neuron subtype. For example, the 10 PV interneurons had substantial cell-to-cell variability in terms of fluorescence change per action potential [Fig. 2(b)]. This may be due to differences in the concentration of calcium indicators, whether through uneven loading of synthetic dyes or differential expression levels of GECIs. There was also variability across the neuron subtypes. In particular, the fluorescence change per action potential was greatest for excitatory neurons, and ∼50% lower for somatostatin-expressing (SST) and PV interneurons [Figs. 2(c) and 2(d)]. These differences are probably due to endogenous calcium buffers in GABAergic interneurons.^44^^,^^45^

Linearity in the spike-to-fluorescence relationship is important. Namely, one action potential should induce the same increase in fluorescence signal, regardless of the baseline calcium level. On average, with the synthetic dye OGB-1, the three cortical neuron subtypes exhibited fluorescence signals that scaled linearly with firing rates [Fig. 2(d)]. For individual neurons, the majority of the PV cells had a linear response curve up to ∼30  Hz, although in a few selected cells, saturation was apparent at high firing rates [Fig. 2(b)]. These observations are consistent with an independent *in vivo* calibration for PV interneurons.^46^ A linear spike-to-fluorescence relationship depends on having a linear calcium-to-fluorescence relationship. Unlike OGB-1, GECIs that rely on cooperative calcium binding, such as GCaMP6, have nonlinear response curves.^23^ If the saturation or nonlinear response profile is known, the distortion may be corrected to some extent in post hoc analysis.^47^

Inferring individual spikes under the high firing rate condition is a challenging problem. Important factors include imaging speed, indicator kinetics, and signal-to-noise ratio. The need for high imaging speed is obvious, as the sampling rate must be faster than the frequency content of the underlying continuous signal to obey the sampling theorem. Indicator kinetics, including rise and decay times, are also crucial. When kinetics is slow relative to spike rate, fluorescence responses to adjacent spikes overlap and cannot be discerned. Signal-to-noise ratio—a factor of the indicator and experimental condition—must be sufficient to detect the fluorescence change due to a single spike. Optimization of these parameters and their interactions is a complex topic, as detailed in a prior study with numerical simulations,^47^ and drives GECI engineering efforts.^23^^,^^48^

## 4 Somatic Calcium Signals: for Measuring a Decrease in Firing Rate

The somatic calcium transient due to an action potential has a near-instantaneous rise and then a relatively long decay. How much does the extended time course obscure the detection of a sudden decrease in firing rate? Figure 2(a) suggests that such decreases should be detectable. In that example, the PV interneuron has a high baseline activity *in vivo*, and fluorescence signals could track fluctuations above and below the median firing rate.

**Fig. 3:**
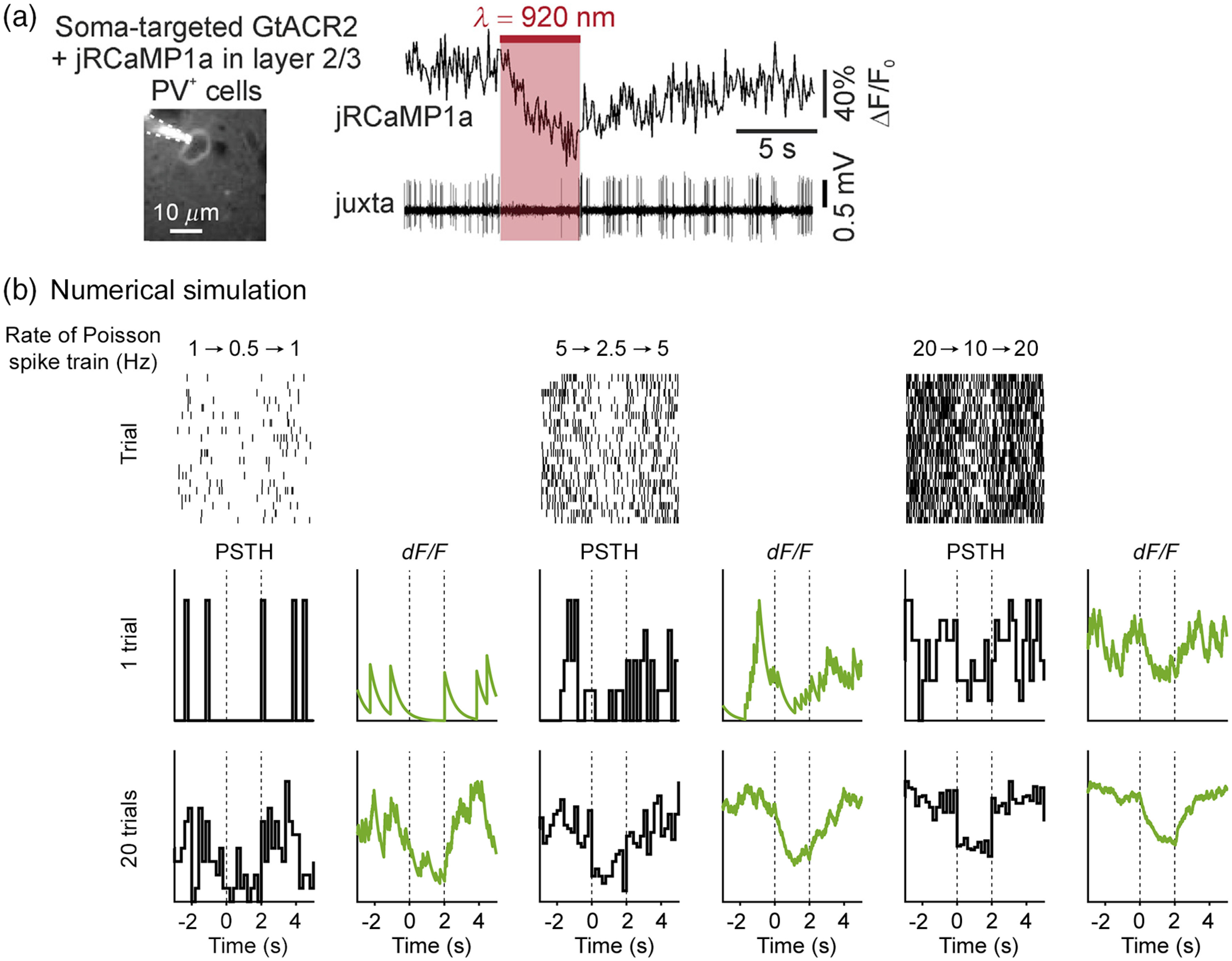
Somatic calcium signals can report a decrease in firing rate. (a) A layer 2/3 PV interneuron expresses the redshifted calcium indicator jRCaMP1a and the soma-targeted opsin GtACR2. Traces show the fluorescence and spiking activity for the neuron *in vivo*. The red-shaded area denoted the time of the illumination to induce inhibition. (b) In these numerical simulations, for each trial, spike times were generated as a Poisson process, in which the rate parameter was halved at t=0  s and then returned to baseline at t=2  s. The calcium response to a spike was approximated as a single-exponential function with a decay time constant of 0.55 s. To determine the fluorescence signal, the spike-induced calcium transients were summed linearly. Peristimulus time histograms (PSTH) were generated by counting spikes in 200-ms bins. The range of the vertical axis for PSTH and fluorescence signals was arbitrary and rescaled for each plot. Panel (a) is adapted from Ref. 49. Panel (b) is unpublished data from the Kwan lab.

There are more explicit demonstrations that calcium signals can report a reduction in firing rate. In one experiment, a PV interneuron co-expresses an opsin for photoinhibition as well as a redshifted calcium sensor *in vivo* [Fig. 3(a)]. The effect of photoinhibition was confirmed by the suppressed spiking activity recorded by a juxtacellular electrode. For a single trial involving a 5-s long silencing period, there was an obvious dip in the calcium signal when spikes were suppressed [red-shaded area, Fig. 3(a)].

A more thorough experimental demonstration of how calcium signals relate to reduced firing rate has been done *in vitro* in acute brain slices (see extended data Fig. 2 in Ref. 50). In particular, that study showed that a decrease in spike rate leads to a negative calcium transient, but hyperpolarization of the membrane potential without any action potential change had no effect on the calcium signal. This is expected because hyperpolarization should have minimal influence on the permeability of the calcium channels in the cell body. In other words, as a measure of inhibition, calcium cannot be used to detect inhibition in the form of hyperpolarization without spiking activity.

The same question can be answered with numerical simulations. For Fig. 3(b), we simulated how the somatic calcium signals would look if spike rate is halved for cells with varying amount of baseline firing. Notably, a reduction in calcium signal is obvious in single trials for neurons with high baseline activity, or by averaging across multiple trials for a wider range of firing rates. The more difficult cases are to discern a decrease in fluorescence if the neuron has low baseline firing, small reduction in spike rate, or only a few trials. However, this problem is not unique to calcium imaging, as peristimulus time histograms for spiking data is also noisy under these conditions [Fig. 3(b)]. And as is the case for spike inference, the sensitivity of detecting a reduction in firing rate would also depend on indicator kinetics and signal-to-noise ratio.

## 5 Axonal Calcium Signals: as a Proxy for Presynaptic Activity

Calcium signals from axonal boutons reflect depolarization in the presynaptic terminals and therefore can provide information about the afferent activity. When applied to long-range projections, axonal calcium imaging has been used to study the flow of information from the primary visual cortex to higher visual areas,^51^ determine the behavioral variables encoded by different afferent inputs to the motor cortex,^52^ and map the spatial organization of long-range feedback projections.^53^

Briefly, membrane depolarization at an axonal terminal leads to calcium influx. For example, in layer 2/3 pyramidal neurons, calcium entry near the presynaptic active zone is mediated by voltage-dependent calcium channels including P/Q- and N-subtypes.^54^^,^^55^ Calcium signals are larger at the synaptic terminals than the flanking axonal segments, presumably due to a nonuniform distribution of calcium channels. The presynaptic calcium influx for a single action potential was estimated to have a fast rise time of ∼1  ms and a decay time constant of ∼60  ms. An axonal calcium transient could be reliably elicited every time by an action potential, with an estimated mean increase of 500 nM at the terminal. Interestingly, when responses to a single action potential were measured across multiple axonal boutons from the same neuron, there was a more than 10-fold variation in the intensity of calcium transients.^56^ This variability across boutons did not depend on distance from the soma but may instead depend on the postsynaptic cell type.^57^ Calcium dynamics in the presynaptic terminals are also influenced by neuromodulators such as adenosine^55^ and dopamine.^58^

**Fig. 4:**
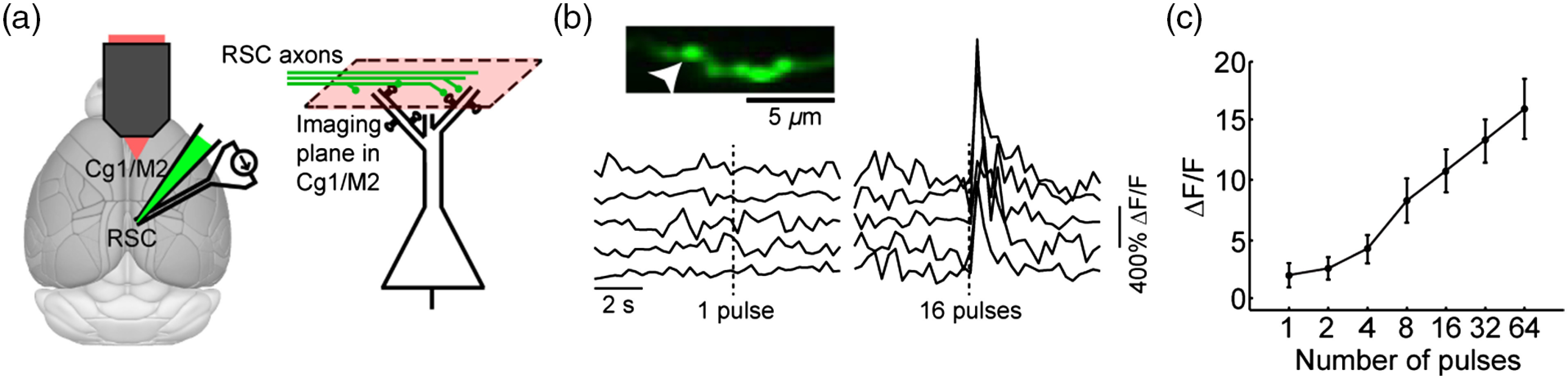
Axonal calcium signals as a function of presynaptic neuronal activation. (a) Schematic of the experimental setup. Viruses were injected to express GCaMP6s in neurons in the RSC. Bipolar stimulating electrodes were implanted also in the RSC. Imaging was done in the Cg1/M2 to visualize the long-range axons from RSC neurons. (b) Top, *in vivo* two-photon image of a GCaMP6s-expressing axonal segment. Arrowhead, the example bouton analyzed. Bottom, ΔF/F traces from the example bouton in response to either 1 or 16 current injection pulses (±150  μA, 10 ms per pulse) per trial. Five trials were shown for each stimulation strength. (c) The trial-averaged ΔF/F response within 1 s of the stimulation as a function of the stimulation strength, for the example bouton in (b). Line, mean±SEM. These are unpublished data from the Kwan lab, related to a recent study.^59^

We have conducted experiments to assess the correlation between axonal calcium levels and afferent activation in an awake mouse^59^ (Fig. 4). For these experiments, we targeted neurons in the retrosplenial cortex (RSC), which send dense axonal projections to the cingulate and secondary motor cortical regions (Cg1/M2).^60^^,^^61^ An adeno-associated virus was used to express GCaMP6s in RSC neurons. We then imaged their axons in Cg1/M2 while simultaneously stimulated RSC using bipolar electrodes [Fig. 4(a)]. In response to raising levels of electrical stimulation, there was a graded increase in the evoked fluorescence transients from axonal boutons [Figs. 4(b) and 4(c)]. The response profile to afferent activation is consistent with a previous *in vitro* calibration done in brain slices (see Supplementary Fig. 2 in Ref. 52). Altogether, these results provide evidence that the calcium signals recorded from axonal compartments *in vivo* is related to depolarization of the afferents.

Because prior study has suggested substantial variability in calcium responses across boutons from the same cell, attempts to use data from a single axonal bouton to infer the firing rate of the presynaptic cell would be prone to large errors. Furthermore, the dependences of the axonal calcium influx on the postsynaptic cell type and neuromodulators mean that an aggregate analysis including many boutons would bias toward subpopulations of terminals with larger calcium amplitudes. These are issues that will affect the interpretations until we gain a deeper understanding of the factors that influence calcium elevations in the axon.

## 6 Axonal Calcium Signals: as a Correlate of Neuromodulator Release

Axonal calcium signals may relate to neuromodulator release, particularly if calcium indicators are expressed in principal cells of a neuromodulatory system. For example, calcium signals can be imaged from cholinergic axons to investigate the role of acetylcholine during active behavior,^62^^,^^63^ from cholinergic and noradrenergic axons to study pupil-related arousal,^64^ and from dopaminergic axons to relate to locomotion and reward-based behavior.^65^ In these examples, neuromodulator release might not have been explicitly inferred, but nevertheless the calcium signals are often interpreted by comparing with previous studies that measure extracellular concentrations of the neuromodulators using methods such as microdialysis or fast-scan cyclic voltammetry.

**Fig. 5:**
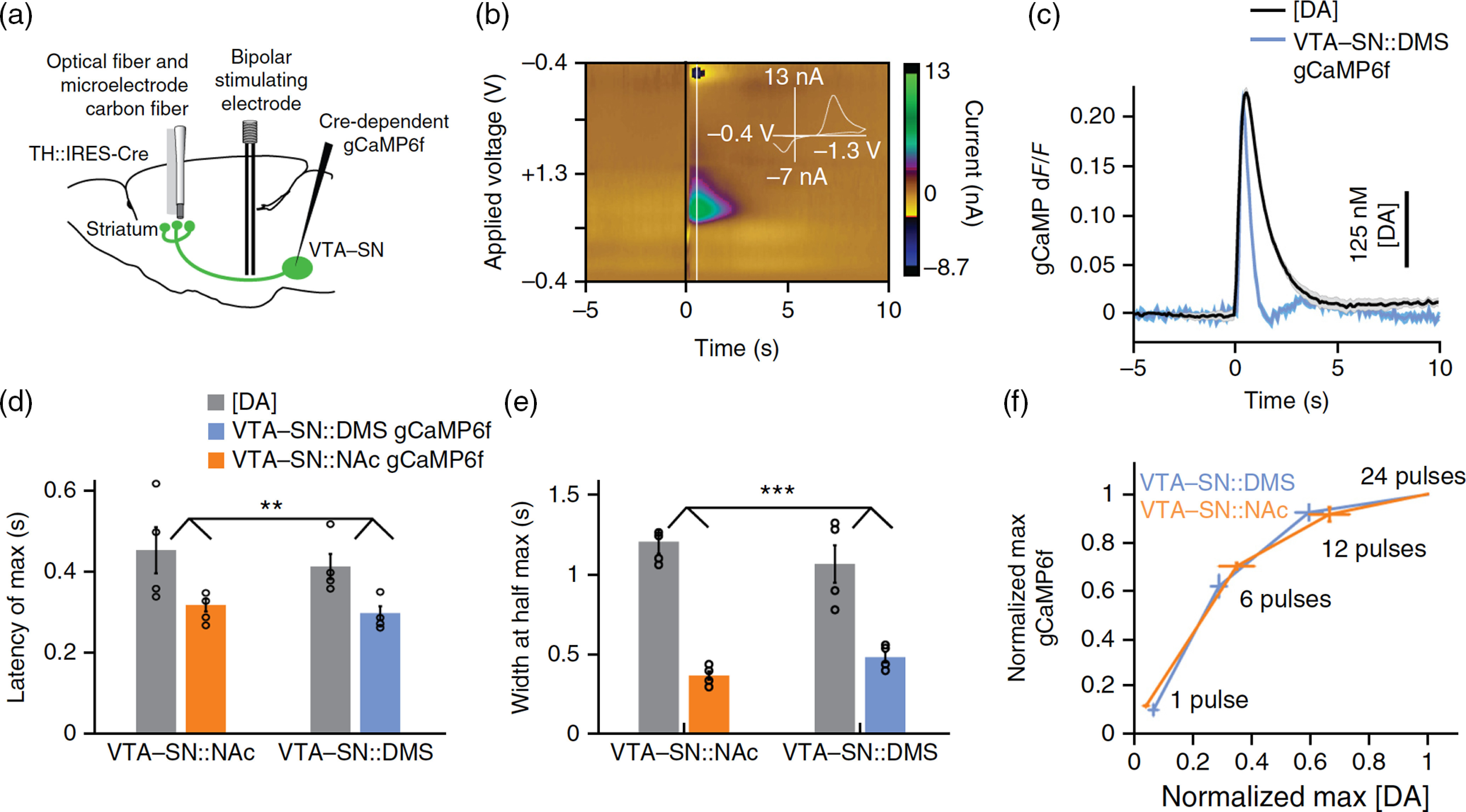
Axonal calcium signals correlate with evoked dopamine release. (a) The medial forebrain bundle was stimulated to evoke dopamine release in a mouse. In the nucleus accumbens (NAc) or dorsomedial striatum (DMS), an optical fiber and a carbon-fiber microelectrode were inserted for photometric measurements of calcium and cyclic voltammetry of extracellular dopamine, respectively. Calcium signals arise from axons of GCaMP6f-expressing dopaminergic neurons in the ventral tegmental area and substantia nigra (VTA-SN). (b) Example recording of evoked dopamine efflux using cyclic voltammetry. (c) Comparison of the trial-averaged axonal calcium signal with the recorded dopamine transient. (d), (e) The latency and width of the calcium and dopamine signals, plotted separately for VTA-SN-to-NAc and VTA-SN-to-DMS axons. (f) The peak calcium signals as a function of the peak evoked dopamine levels for various stimulation strengths. Figure is adapted from Ref. 66. Reproduced with permission, courtesy of Springer Nature.

At least one study has empirically tested the relation between calcium signals and evoked neuromodulator release.^66^ Stimulation of the medial forebrain bundle leads to the release of dopamine in the striatum. This is an ideal testbed because the evoked dopamine transient is large. In this study, a carbon-fiber microelectrode was inserted into the striatum for cyclic voltammetry measurements of extracellular dopamine concentration [Figs. 5(a) and 5(b)]. Simultaneously, an optical fiber was inserted nearby to record the summed fluorescence signal of many dopaminergic axonal fibers. Relative to the evoked elevation of dopamine, the fluorescence transient had a shorter latency to peak, and a narrower width at half maximum [Figs. 5(c)–5(e)]. Importantly, calcium signals had a decent dynamic range. That is, fluorescence was sensitive to a range of evoked dopamine concentrations, and only began to saturate at the highest stimulation strengths for the largest evoked dopamine transients [Fig. 5(f)].

There are a number of caveats relating axonal calcium signals to neuromodulator release. Figure 5 shows that the calcium signals can track rapid phasic changes in dopamine concentration; however, the tonic level may not be detectable, as the calcium indicator can report relative changes but not the absolute amount of axonal activity. Furthermore, although an action potential would reliably depolarize the presynaptic terminal and cause calcium influx, the next steps in the chain of events involve the probabilistic release of a synaptic vesicle, and then the discharge and diffusion of several thousand neuromodulator molecules from a vesicle into extracellular space. As a result, the inference of neuromodulator levels from axonal calcium signals is complicated by additional, stochastic steps. Thus, although *in vivo* evidence suggests that the inference is reasonable when averaging across many axons and numerous trials, the correlation is likely much weaker at the single-axon level.

## 7 Dendritic Calcium Signals: as a Readout of Backpropagating Action Potentials

In dendrites, membrane depolarization can reflect a variety of passive and active electrical processes.^17^^,^^18^ One important source of dendritic calcium elevations is the backpropagating action potential (bAP). A bAP occurs because an action potential initiated close to the soma can travel bidirectionally, such that it would not only continue along the axon but also propagate backward into the dendritic tree. As a retrograde signal of neuronal output to the dendritic tree, the bAP may play important roles in synaptic plasticity and associative learning.^67^

Propagation of the bAP relies on active conductances including voltage-activated sodium channels.^68^ In the absence of synaptic inputs, calcium can enter the dendritic shafts and spines through voltage-gated calcium channels.^54^ The channel subtypes involved vary depending on the cell type.^17^ The bAP is present in a variety of cell types, such as cortical pyramidal neurons,^68^ cortical GABAergic bitufted interneurons,^69^ and striatal medium spiny neurons,^70^ but is much attenuated in other cell types, such as cerebellar Purkinje cells.^71^

**Fig. 6:**
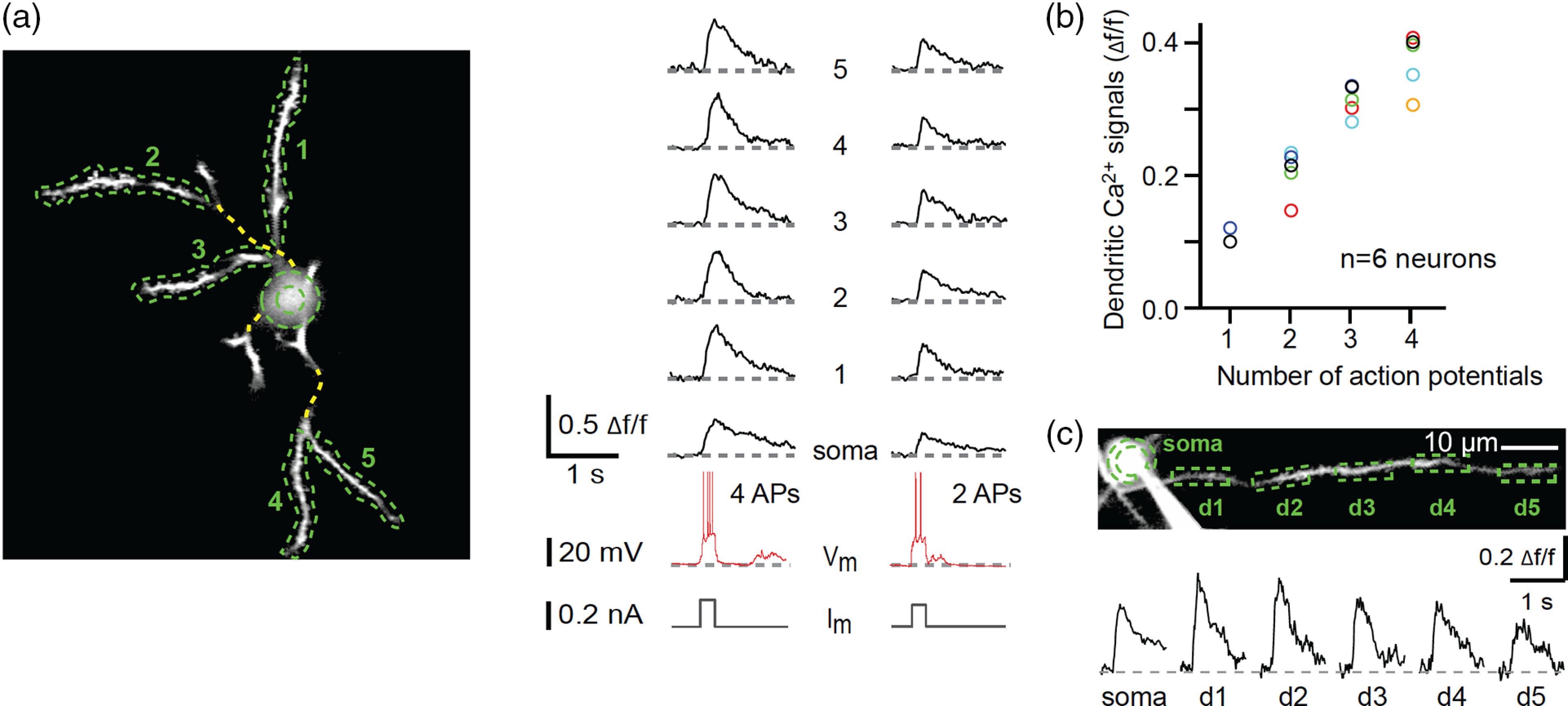
Dendritic calcium signals from bAPs. (a) The left panel shows an *in vivo* two-photon image of a layer 2/3 neuron in the visual cortex loaded with OGB-1. The cell was also targeted for whole-cell recording. Green lines denote the dendritic regions analyzed. The right panel shows fluorescence traces for each of the dendritic region in response to somatic current injection producing either four or two action potentials. (b) The mean amplitude of dendritic calcium signals versus the number of evoked action potentials. Each circle denotes a cell. (c) Fluorescence responses in a dendrite as a function of distance from soma. Figure is adapted from Ref. 77. Reproduced with permission, courtesy of Springer Nature.

Experiments that combine imaging with electrophysiology have demonstrated dendritic calcium transients that are coincident with action potentials recorded in the soma.^72^^,^^73^ The more ideal and causal test would be to elicit action potentials by injecting current into the soma.^74^^–^^76^ In one study,^77^ in an anesthetized mouse, a layer 2/3 neuron in the visual cortex was targeted for whole-cell recording while a two-photon microscope monitors fluorescent signals in the proximal dendritic segments [Fig. 6(a)]. Action potentials were evoked by current injection into the cell body. Although the bAP is around a millisecond in duration, the fluorescent transient is prolonged due to the life cycle of calcium and indicator kinetics. The fluorescent signals were linearly associated with the number of action potentials [Fig. 6(b)]. The bAP-associated calcium signal spread along the proximal dendrite with noticeable attenuation at the farthest imaged locations [Fig. 6(c)].

The amount of bAP-associated calcium influx is dependent on a number of factors. One important factor is the distance from the initiation site of the action potential. In anesthetized animals, for layer 2/3 pyramidal neurons, the bAP attenuates and broadens as a function of distance from the soma, such that the calcium elevation becomes negligible beyond 200 to 250  μm.^74^^,^^75^ For layer 5 pyramidal neurons, beyond the main bifurcation point of the apical tuft, calcium influx is absent or unreliable for single bAP.^76^ Distance is the determining factor, because unlike the apical tuft, there is considerable correlation between somatic firing and calcium transients in the more proximal apical trunk and in the basal dendrites of these neurons.^73^ Other factors include differences in the morphology of the dendritic tree^78^ or the distribution of dendritic voltage-gated channels.

## 8 Dendritic Calcium Signals: as a Readout of Dendritic Regenerative Events

A regenerative event may be initiated at the dendrites in response to the temporally synchronous and spatially clustered activation of many synaptic inputs. The resulting broad dendritic spike is accompanied by large-amplitude calcium influx. Calcium entry may be mediated through N-methyl-d-aspartate (NMDA)-type glutamate receptors and voltage-gated calcium channels. Their relative contribution is thought to depend on the dendritic location.^79^^,^^80^ For example, in layer 5 pyramidal neurons *in vitro*, calcium elevations from dendritic spikes could be confined to the activated branch in the most distal, fine tuft dendrites for NMDA spikes^79^ or spread to ∼50 to 150 *μ*m from the initiation site in the apical tuft near the main bifurcation point for calcium spikes.^81^^–^^83^ Unlike sodium action potentials, the depolarization from calcium spikes can last hundreds of milliseconds.

**Fig. 7:**
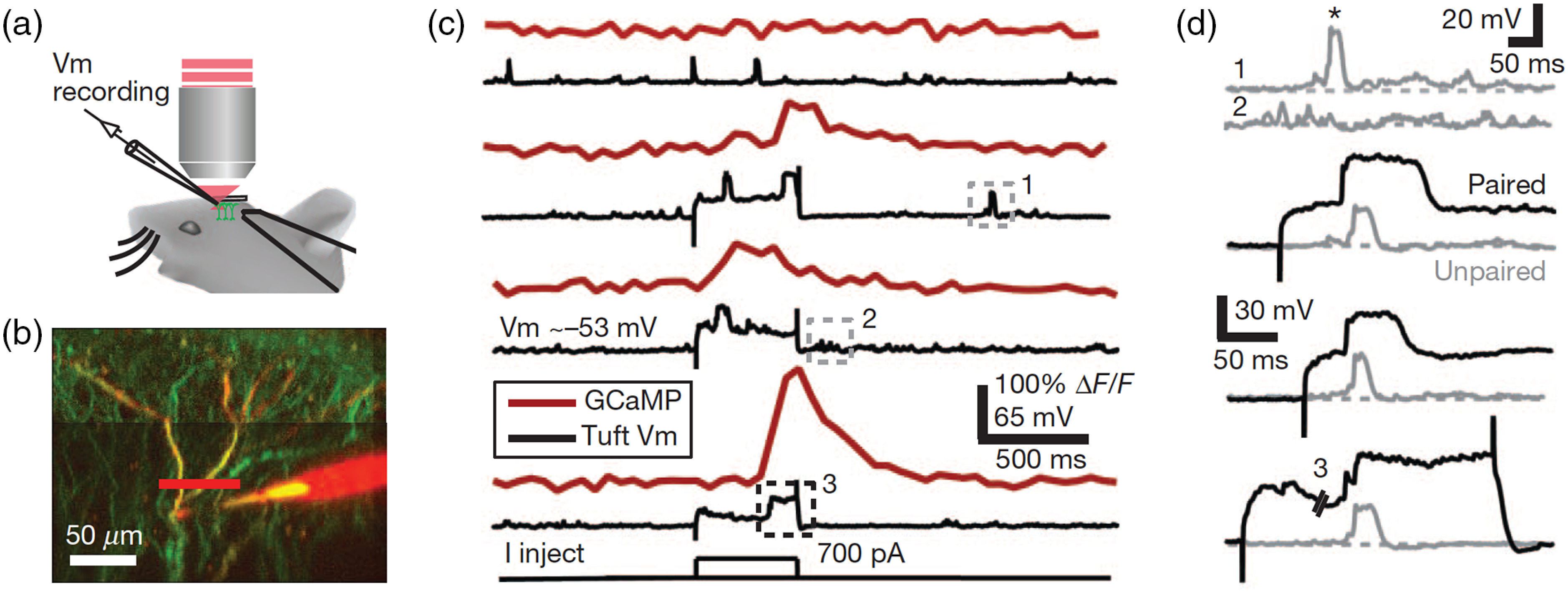
Dendritic calcium signals from dendritic regenerative events. (a) Schematic of the experiment involving *in vivo* two-photon imaging and dendritic patch-clamp recording in an anesthetized mouse. (b) A layer 5 neuron in the barrel cortex expresses GCaMP3 (green). The patch pipette was filled with Alexa Fluor 594 (red). (c) The membrane potential at the apical dendrite (black) was plotted along with calcium signals (red) imaged in line-scan mode as indicated by the red line in (b). The top voltage trace is from a trial without current injection. The lower three voltage traces are from trials with a 500-ms, 700-pA current step. (d) Magnified view of the dashed boxes in (c). The gray traces are spontaneous dendritic voltage events. The black traces show plateau potentials during the paired events in which current was injected. Figure is adapted from Ref. 84. Reproduced with permission, courtesy of Springer Nature.

Simultaneous calcium imaging and dendritic patch-clamp recording of layer 5 pyramidal neurons have provided evidence for compartmentalized calcium elevations that are regulated locally in dendrites *in vivo*. An early *in vivo* study showed that dendritic calcium events and somatic action potentials are not always coupled—namely, sometimes there is somatic burst spiking but no detectable change in dendritic calcium, whereas other times there is only one spike but a large dendritic calcium transient near the main bifurcation point of the apical dendrite.^76^ In a more recent study,^84^ it was shown that moderate dendritic voltage events in the apical trunk were not associated with any detectable calcium signals in the distal tuft dendrites in an anesthetized mouse [boxes 1 and 2 in Figs. 7(a)–7(d)]. However, when a depolarizing current step was injected at the apical trunk, the coincident arrival of a spontaneous voltage event would induce a longer-lasting plateau depolarization, which was accompanied by a large-amplitude calcium transient in tuft dendrites [box 3 in Figs. 7(c) and 7(d)]. Critically, the current step alone was not sufficient to induce detectable calcium signals in the distal tuft. The interpretation for these data is that the dendritic calcium transients were due to occurrences of regenerative plateau potentials in the apical compartment, on the basis of the stimulation location. Intriguingly, this study presented relatively widespread dendritic calcium signals across multiple distal tuft branches, which differs from a couple of other accounts of transients that are typically more spatially restricted^73^^,^^85^ or branch-specific.^86^ One challenge is that without the knowledge of the calcium signals in the apical trunk, some of the putative branch-specific signals may in fact be more global plateau potentials that have not invaded all branches, and thus masquerade as compartmentalized calcium transients. A recent study estimated only about 15% of the calcium signals in distal tufts are independent from those in the apical trunk in layer 5 pyramidal neurons during awake behavior.^87^ Pharmacological manipulations could provide additional clues as to the origin of these dendritic calcium signals *in vivo*.^85^^,^^88^ Nevertheless, more work is needed to unequivocally attribute *in vivo* dendritic calcium transients to specific types of nonlinear regenerative events.

## 9 Dendritic Calcium Signals: as a Readout of Synaptic Input

Dendritic spines are protrusions on the dendritic shaft. Most dendritic spines are functional glutamatergic synapses that receive excitatory inputs. Upon depolarization from an excitatory input, calcium ions enter the spine compartment with minimal outflow to the dendritic shaft.^89^^,^^90^ The localized signal is unlike the situation for bAP in which calcium invades both spines and the connecting shaft.^54^ For pyramidal neurons, a large portion of the synaptically evoked calcium influx is probably driven by NMDA receptors,^54^^,^^91^ with some contributions by voltage-gated calcium channels.^92^^,^^93^ Because the localized calcium transient is a correlate of synaptic activation, spine imaging *in vivo* is a powerful method to characterize the organization of synaptic inputs, for example, to understand how inputs are integrated to give rise to feature selectivity in visual cortical neurons.^77^^,^^94^

**Fig. 8:**
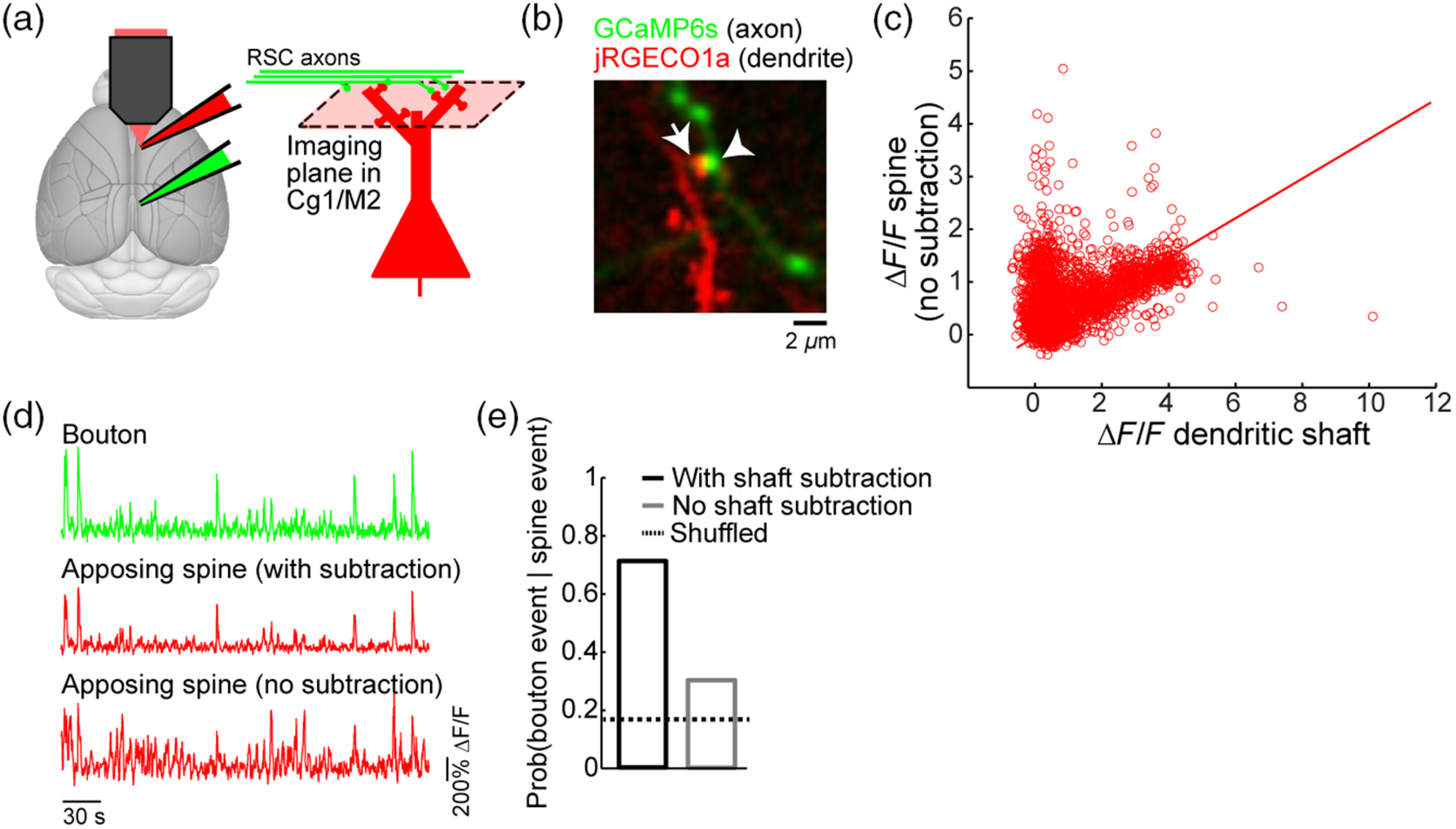
Dendritic calcium signals from synaptic inputs. (a) The experimental setup involving expression of GCaMP6s in RSC and jRGECO1a in Cg1/M2. (b) An *in vivo* two-photon image of GCaMP6s-expressing axonal boutons and jRGECO1a-expressing dendrites in Cg1/M2. (c) A scatter plot of the fluorescence transients (ΔF/F) measured from spine indicated by arrow in (b) against the ΔF/F measured from the adjacent dendritic shaft. Each open circle represents an image frame. Line, a least-squares regression line forced through the origin. (d) Fluorescence traces for the bouton and apposing spine (either with subtraction or no subtraction of the shaft contribution) in (b). (e) The probability of detecting a presynaptic calcium event in the bouton within ±0.276  s (i.e., ± the duration of one image frame) given a postsynaptic calcium event in the apposing spine, either with subtraction or no subtraction of the shaft contribution. Calcium events were determined from fluorescence traces using a peeling algorithm. Shuffled level was calculated by randomly shuffling the calcium event times of the bouton, and averaged across 100 replicates. These are unpublished data from the Kwan lab.

Because subthreshold synaptic activation—one that occurs in the absence of somatic action potentials or other regenerative events—induces only localized influx, any calcium transient detected in the shaft and spine at the same time is likely due to other mechanisms such as bAP or dendritic spikes. Therefore, a common practice is to isolate the local component by removing those fluorescent transients that co-occur in the shaft and spine in *post hoc* analysis using a subtraction procedure.^6^^,^^95^^,^^96^ As an additional benefit, noises that cofluctuate in the shaft and spine, for example, from motion artifact, would be reduced by the subtraction procedure. How well does the procedure work? To answer this question, we have imaged spontaneous calcium transients in apposing bouton-spine pairs in awake mice. More specifically, we expressed GCaMP6s in RSC excitatory neurons and the redshifted calcium sensor jRGEOC1a in Cg1/M2 neurons [unpublished data; Fig. 8(a)]. In Cg1/M2, putative connected bouton-spine pairs could be identified based on proximity [Fig. 8(b)]. We followed a typical subtraction procedure, by using linear regression to estimate the shaft-spine signal ratio, which allows us to scale the shaft signal and subtract its contribution from the spine signal [Fig. 8(c)]. As expected for functional synapses, the subtraction-isolated, local fluorescent transients from the dendritic spine were highly correlated with signals from the axonal bouton [Fig. 8(d)]. To quantify the relationship, we converted the fluorescence transients into calcium events using a peeling algorithm,^47^ and then calculated the conditional probability of observing a presynaptic event given a postsynaptic event. In an example bouton–spine pair, we estimated ∼70% of the postsynaptic events imaged in the dendritic spine were coincident with synaptic input [Fig. 8(e)]. By contrast, the estimate fell to ∼30% when the subtraction procedure was not used, suggesting greater nonsynaptic contributions to the spine calcium signal. Isolation of the local component may be improved if the regression considers the distinct calcium diffusion and decay characteristics in the shaft and spine compartments.^87^

One caveat of the aforementioned subtraction method is that, by definition, the procedure removes synaptic inputs that correlate with the occurrences of dendritic spikes and bAPs. Arguably those may include the strongest and clustered patterns of inputs that affect spiking activity and synaptic plasticity. To mitigate this issue, synaptic calcium signals may be imaged in the absence of spiking by hyperpolarizing the cell body or usingpharmacology.^77^^,^^97^ This would prevent bAP, although dendritic regenerative events may still occur in the distal compartment due to the space-clamp problem. Another caveat is that calcium enters dendritic spines predominantly through NMDAR, so we would report only those synaptic activations that are sufficiently strong to relieve the magnesium block. Because of these challenges in detecting the strongest and weakest activations, synaptic calcium signals are expected to report a fraction of the synaptic inputs arriving at a dendritic spine and should be interpreted accordingly.

## 10 Putting It All Together

From the viewpoint of an experimenter, current imaging techniques provide a limited picture of the entire neuronal structure. We typically visualize either the somatic, axonal, or dendritic compartment, and therefore it has been convenient to categorize calcium signals based on the imaging location. However, this classification ignores the dynamic nature of the physiology. Instead, if we consider from the vantage point of a neuron, a sodium action potential initiated near the soma would induce calcium influx in the cell body, and then would propagate and elevate calcium also in the axon and proximal dendrite. Or take the case of synaptic inputs, which initially elevate calcium in spines, but together a strong barrage of inputs could lead to a regenerative event that is accompanied by more widespread dendritic calcium signals.^98^^,^^99^ In a more complex situation, for example, in layer 5 pyramidal neurons, subthreshold synaptic inputs may pair with a bAP to drive plateau potentials and burst firing at the soma^100^—a sequence of events that is expected to induce calcium influx in almost all compartments of a neuron.

Presumably the more complex sequences of physiological events are more prevalent during active behavior. This is because the strength of regenerative events such as the bAP is boosted by synaptic inputs, which are more frequent and powerful *in vivo* and in awake conditions.^101^^,^^102^ GABAergic neurons have complex firing patterns *in vivo*, and the strengthening or relief of inhibition could strongly influence calcium spikes^103^ and control calcium influx into dendritic spines.^104^ Indeed, dendritic calcium signals were found to be substantially enhanced in the same animal when awake rather than anesthetized.^105^ Putting it all together, instead of considering calcium elevations from isolable events in various compartments, one can move toward using the overall spatiotemporal pattern of calcium signals across the entire cell to infer the underlying spatiotemporal dynamics of electrical depolarization in the neuron.

## 11 Looking Ahead

We need methods that can uncover calcium signals over a broader spatial scale. Some techniques, such as fiber photometry, provide no optical sectioning. Because calcium signals could come from a mix of somatic, axonal, and dendritic sources, the neural correlates should be interpreted with caution. One improvement is to use genetic strategies to restrict expression to the soma^106^ or axons,^107^ such that calcium indicators reside in only a specific cellular compartment; however, experiments would still suffer from the same problems as discussed in the last section.

To increase the spatial extent of imaging, emerging technologies enable data acquisition across multiple axial planes or from a volume. In this review article, most of the examples were acquired with two-photon microscopes. Using a high numerical-aperture objective, the lateral resolution of this method is ∼0.5  μm and axial resolution is ∼2  μm,^108^ sufficient to visualize small compartments such as single dendritic spines. To maintain this spatial resolution but now over greater spatial extent, two-photon microscopes have incorporated design elements such as electrically tunable lens,^109^ Bessel focus,^110^ and remote focusing.^87^ Studies using these new microscopes have demonstrated rapid imaging of multiple neuronal compartments, such as numerous dendritic tuft branch locations along with the apical dendritic trunk or the cell body.

As the ability to report calcium transients and infer electrical events depends on the properties of the fluorescent indicator, the choice of the indicator should be matched to the needs of the experiment. For the latest generation of GECIs, this is exactly the rationale behind the multiple variants of the indicators.^48^^,^^111^ For example, the most sensitive variant is useful for the detection of single action potentials in the soma. By contrast, a variant with less sensitivity but brighter baseline fluorescence is desirable for dendritic imaging, because the higher basal signal is helpful for confirming the presence of a spine.

The indicator development, together with new imaging technologies, promises to deliver the full richness of calcium dynamics in neurons. This will help with addressing the current limitations of the calcium imaging technique and enable more accurate interpretations of the results.

## References

[1] Takahashi N. et al., “Active cortical dendrites modulate perception,” Science 354(6319), 1587–1590 (2016). SCIEAS 0036-8075 10.1126/science.aah6066 28008068

[2] Makino H. et al., “Transformation of cortex-wide emergent properties during motor learning,” Neuron 94(4), 880–890.e8 (2017). NERNET 0896-6273 10.1016/j.neuron.2017.04.015 28521138 PMC5502752

[3] Siniscalchi M. J. et al., “Fast and slow transitions in frontal ensemble activity during flexible sensorimotor behavior,” Nat. Neurosci. 19(9), 1234–1242 (2016). NANEFN 1097-6256 10.1038/nn.4342 27399844 PMC5003707

[4] Ackman J. B. Burbridge T. J. Crair M. C., “Retinal waves coordinate patterned activity throughout the developing visual system,” Nature 490(7419), 219–225 (2012). 10.1038/nature11529 23060192 PMC3962269

[5] de Vries S. E. J. et al., “A large-scale, standardized physiological survey reveals higher order coding throughout the mouse visual cortex,” bioRxiv 359513 (2019). 10.1101/359513

[6] Chen T. W. et al., “Ultrasensitive fluorescent proteins for imaging neuronal activity,” Nature 499(7458), 295–300 (2013). 10.1038/nature12354 23868258 PMC3777791

[7] Dana H. et al., “Sensitive red protein calcium indicators for imaging neural activity,” Elife 5, e12727 (2016). 10.7554/eLife.12727 27011354 PMC4846379

[8] Daigle T. L. et al., “A suite of transgenic driver and reporter mouse lines with enhanced brain-cell-type targeting and functionality,” Cell 174(2), 465–480.e22 (2018). CELLB5 0092-8674 10.1016/j.cell.2018.06.035 30007418 PMC6086366

[9] Denk W. Strickler J. H. Webb W. W., “Two-photon laser scanning fluorescence microscopy,” Science 248(4951), 73–76 (1990). SCIEAS 0036-8075 10.1126/science.2321027 2321027

[10] Horton N. G. et al., “In vivo three-photon microscopy of subcortical structures within an intact mouse brain,” Nat. Photonics 7(3), 205–209 (2013). NPAHBY 1749-4885 10.1038/nphoton.2012.336 PMC3864872 24353743

[11] Stirman J. N. et al., “Wide field-of-view, multi-region, two-photon imaging of neuronal activity in the mammalian brain,” Nat. Biotechnol. 34(8), 857–862 (2016). NABIF9 1087-0156 10.1038/nbt.3594 27347754 PMC4980167

[12] Neher E. Augustine G. J., “Calcium gradients and buffers in bovine chromaffin cells,” J. Physiol. 450, 273–301 (1992). JPHYA7 0022-3751 10.1113/jphysiol.1992.sp019127 1331424 PMC1176122

[13] Helmchen F. Imoto K. Sakmann B., “${\mathrm{Ca}}^{2+}$ buffering and action potential-evoked ${\mathrm{Ca}}^{2+}$ signaling in dendrites of pyramidal neurons,” Biophys. J. 70(2), 1069–1081 (1996). BIOJAU 0006-3495 10.1016/S0006-3495(96)79653-4 8789126 PMC1225009

[14] Grienberger C. Konnerth A., “Imaging calcium in neurons,” Neuron 73(5), 862–885 (2012). NERNET 0896-6273 10.1016/j.neuron.2012.02.011 22405199

[r15] Grewe B. F. Helmchen F., “Optical probing of neuronal ensemble activity,” Curr. Opin. Neurobiol. 19(5), 520–529 (2009). COPUEN 0959-4388 10.1016/j.conb.2009.09.003 19854041

[16] Peron S. Chen T. W. Svoboda K., “Comprehensive imaging of cortical networks,” Curr. Opin. Neurobiol. 32, 115–123 (2015). COPUEN 0959-4388 10.1016/j.conb.2015.03.016 25880117

[17] Higley M. J. Sabatini B. L., “Calcium signaling in dendrites and spines: practical and functional considerations,” Neuron 59(6), 902–913 (2008). NERNET 0896-6273 10.1016/j.neuron.2008.08.020 18817730

[r18] Grienberger C. Chen X. Konnerth A., “Dendritic function in vivo,” Trends Neurosci. 38(1), 45–54 (2015). TNSCDR 0166-2236 10.1016/j.tins.2014.11.002 25432423

[r19] Augustine G. J. Santamaria F. Tanaka K., “Local calcium signaling in neurons,” Neuron 40(2), 331–346 (2003). NERNET 0896-6273 10.1016/S0896-6273(03)00639-1 14556712

[20] Augustine G. J. Charlton M. P. Smith S. J., “Calcium action in synaptic transmitter release,” Annu. Rev. Neurosci. 10, 633–693 (1987). ARNSD5 0147-006X 10.1146/annurev.ne.10.030187.003221 2436546

[21] Tsien R. Y., “Monitoring cell calcium” in Calcium as Cellular Regulator, Carafoli E. Klee C., Eds., Oxford University Press, Oxford (1999).

[r22] Helmchen F. Tank D. W., “A single-compartment model of calcium dynamics in nerve terminals and dendrites,” Cold Spring Harb. Protoc. 2015(2), 155–167 (2015). 10.1101/pdb.top085910 25646507

[23] Rose T. et al., “Putting a finishing touch on GECIs,” Front. Mol. Neurosci. 7, 88 (2014). 10.3389/fnmol.2014.00088 25477779 PMC4235368

[24] Stringer C. Pachitariu M., “Computational processing of neural recordings from calcium imaging data,” Curr. Opin. Neurobiol. 55, 22–31 (2019). COPUEN 0959-4388 10.1016/j.conb.2018.11.005 30530255

[25] Siniscalchi M. J. Wang H. Kwan A. C., “Enhanced population coding for rewarded choices in the medial frontal cortex of the mouse,” Cereb. Cortex bhy292 (2019). 53OPAV 1047-3211 10.1093/cercor/bhy292 PMC6735259 30615132

[26] Ohki K. et al., “Functional imaging with cellular resolution reveals precise micro-architecture in visual cortex,” Nature 433(7026), 597–603 (2005). 10.1038/nature03274 15660108

[27] Makino H. Komiyama T., “Learning enhances the relative impact of top-down processing in the visual cortex,” Nat. Neurosci. 18(8), 1116–1122 (2015). NANEFN 1097-6256 10.1038/nn.4061 26167904 PMC4523093

[28] Markram H. Helm P. J. Sakmann B., “Dendritic calcium transients evoked by single back-propagating action potentials in rat neocortical pyramidal neurons,” J. Physiol. 485(Pt. 1), 1–20 (1995). JPHYA7 0022-3751 10.1113/jphysiol.1995.sp020708 7658365 PMC1157968

[29] Schiller J. Helmchen F. Sakmann B., “Spatial profile of dendritic calcium transients evoked by action potentials in rat neocortical pyramidal neurones,” J. Physiol. 487(Pt. 3), 583–600 (1995). JPHYA7 0022-3751 10.1113/jphysiol.1995.sp020902 8544123 PMC1156647

[30] Neher E., “Details of ${\mathrm{Ca}}^{2+}$ dynamics matter,” J. Physiol. 586(8), 2031–2031 (2008). JPHYA7 0022-3751 10.1113/jphysiol.2008.153080 18413335 PMC2465202

[31] Kerr J. N. Greenberg D. Helmchen F., “Imaging input and output of neocortical networks in vivo,” Proc. Natl. Acad. Sci. U. S. A. 102(39), 14063–14068 (2005). PNASA6 0027-8424 10.1073/pnas.0506029102 16157876 PMC1201343

[r32] Kwan A. C. Dan Y., “Dissection of cortical microcircuits by single-neuron stimulation in vivo,” Curr. Biol. 22(16), 1459–1467 (2012). CUBLE2 0960-9822 10.1016/j.cub.2012.06.007 22748320 PMC3467311

[33] Tian L. et al., “Imaging neural activity in worms, flies and mice with improved GCaMP calcium indicators,” Nat. Methods 6(12), 875–881 (2009).1548-7091 10.1038/nmeth.1398 19898485 PMC2858873

[34] Vogelstein J. T. et al., “Spike inference from calcium imaging using sequential Monte Carlo methods,” Biophys. J. 97(2), 636–655 (2009). BIOJAU 0006-3495 10.1016/j.bpj.2008.08.005 19619479 PMC2711341

[35] Grewe B. F. et al., “High-speed in vivo calcium imaging reveals neuronal network activity with near-millisecond precision,” Nat. Methods 7(5), 399–405 (2010).1548-7091 10.1038/nmeth.1453 20400966

[36] Fries P., “Modulation of oscillatory neuronal synchronization by selective visual attention,” Science 291(5508), 1560–1563 (2001). SCIEAS 0036-8075 10.1126/science.1055465 11222864

[37] Kwan A. C. et al., “Spatiotemporal dynamics of rhythmic spinal interneurons measured with two-photon calcium imaging and coherence analysis,” J. Neurophysiol. 104(6), 3323–3333 (2010). JONEA4 0022-3077 10.1152/jn.00679.2010 20861442 PMC3007658

[38] Lin B. J. Chen T. W. Schild D., “Cell type-specific relationships between spiking and [${\mathrm{Ca}}^{2+}$]i in neurons of the Xenopus tadpole olfactory bulb,” J. Physiol. 582(Pt. 1), 163–175 (2007). JPHYA7 0022-3751 10.1113/jphysiol.2006.125963 17463049 PMC2075311

[39] Moreaux L. Laurent G., “Estimating firing rates from calcium signals in locust projection neurons in vivo,” Front. Neural Circuits 1, 2 (2007). 10.3389/neuro.04.002.2007 18946544 PMC2526277

[40] Kerlin A. M. et al., “Broadly tuned response properties of diverse inhibitory neuron subtypes in mouse visual cortex,” Neuron 67(5), 858–871 (2010). NERNET 0896-6273 10.1016/j.neuron.2010.08.002 20826316 PMC3327881

[41] Kamigaki T. Dan Y., “Delay activity of specific prefrontal interneuron subtypes modulates memory-guided behavior,” Nat. Neurosci. 20(6), 854–863 (2017). NANEFN 1097-6256 10.1038/nn.4554 28436982 PMC5554301

[42] Kim D. et al., “Distinct roles of parvalbumin- and somatostatin-expressing interneurons in working memory,” Neuron 92(4), 902–915 (2016). NERNET 0896-6273 10.1016/j.neuron.2016.09.023 27746132

[43] Kim H. et al., “Prefrontal parvalbumin neurons in control of attention,” Cell 164(1–2), 208–218 (2016). CELLB5 0092-8674 10.1016/j.cell.2015.11.038 26771492 PMC4715187

[44] Lee S. H. et al., “Differences in ${\mathrm{Ca}}^{2+}$ buffering properties between excitatory and inhibitory hippocampal neurons from the rat,” J. Physiol. 525(2), 405–418 (2000). JPHYA7 0022-3751 10.1111/tjp.2000.525.issue-2 10835043 PMC2269951

[45] Aponte Y. Bischofberger J. Jonas P., “Efficient ${\mathrm{Ca}}^{2+}$ buffering in fast-spiking basket cells of rat hippocampus,” J. Physiol. 586(8), 2061–2075 (2008). JPHYA7 0022-3751 10.1113/jphysiol.2007.147298 18276734 PMC2465201

[46] Hofer S. B. et al., “Differential connectivity and response dynamics of excitatory and inhibitory neurons in visual cortex,” Nat. Neurosci. 14(8), 1045–1052 (2011). NANEFN 1097-6256 10.1038/nn.2876 21765421 PMC6370002

[47] Lutcke H. et al., “Inference of neuronal network spike dynamics and topology from calcium imaging data,” Front. Neural Circuits 7, 201 (2013). 10.3389/fncir.2013.00201 24399936 PMC3871709

[48] Inoue M. et al., “Rational engineering of XCaMPs, a multicolor GECI suite for in vivo imaging of complex brain circuit dynamics,” Cell 177, 1346–1360.e24 (2019). CELLB5 0092-8674 10.1016/j.cell.2019.04.007 31080068

[49] Forli A. et al., “Two-photon bidirectional control and imaging of neuronal excitability with high spatial resolution in vivo,” Cell Rep. 22(11), 3087–3098 (2018). 10.1016/j.celrep.2018.02.063 29539433 PMC5863087

[50] Otis J. M. et al., “Prefrontal cortex output circuits guide reward seeking through divergent cue encoding,” Nature 543(7643), 103–107 (2017). 10.1038/nature21376 28225752 PMC5772935

[51] Glickfeld L. L. et al., “Cortico-cortical projections in mouse visual cortex are functionally target specific,” Nat. Neurosci. 16(2), 219–226 (2013). NANEFN 1097-6256 10.1038/nn.3300 23292681 PMC3808876

[52] Petreanu L. et al., “Activity in motor-sensory projections reveals distributed coding in somatosensation,” Nature 489(7415), 299–303 (2012). 10.1038/nature11321 22922646 PMC3443316

[53] Marques T. et al., “The functional organization of cortical feedback inputs to primary visual cortex,” Nat. Neurosci. 21(5), 757–764 (2018). NANEFN 1097-6256 10.1038/s41593-018-0135-z 29662217

[54] Koester H. J. Sakmann B., “Calcium dynamics in single spines during coincident pre- and postsynaptic activity depend on relative timing of back-propagating action potentials and subthreshold excitatory postsynaptic potentials,” Proc. Natl. Acad. Sci. U. S. A. 95(16), 9596–9601 (1998). PNASA6 0027-8424 10.1073/pnas.95.16.9596 9689126 PMC21384

[55] Cox C. L. et al., “Action potentials reliably invade axonal arbors of rat neocortical neurons,” Proc. Natl. Acad. Sci. U. S. A. 97(17), 9724–9728 (2000). PNASA6 0027-8424 10.1073/pnas.170278697 10931955 PMC16932

[56] Koester H. J. Sakmann B., “Calcium dynamics associated with action potentials in single nerve terminals of pyramidal cells in layer 2/3 of the young rat neocortex,” J. Physiol. 529(Pt. 3), 625–646 (2000). JPHYA7 0022-3751 10.1111/tjp.2000.529.issue-3 11118494 PMC2270226

[57] Koester H. J. Johnston D., “Target cell-dependent normalization of transmitter release at neocortical synapses,” Science 308(5723), 863–866 (2005). SCIEAS 0036-8075 10.1126/science.1100815 15774725

[58] Burke K. J. Jr. Keeshen C. M. Bender K. J., “Two forms of synaptic depression produced by differential neuromodulation of presynaptic calcium channels,” Neuron 99(5), 969–984.e7 (2018). NERNET 0896-6273 10.1016/j.neuron.2018.07.030 30122380 PMC7874512

[59] Ali F. et al., “Ketamine disinhibits dendrites and enhances calcium signals in prefrontal dendritic spines,” bioRxiv 659292 (2019). 10.1101/659292 PMC6946708 31911591

[60] Yamawaki N. Radulovic J. Shepherd G. M., “A corticocortical circuit directly links retrosplenial cortex to M2 in the mouse,” J. Neurosci. 36(36), 9365–9374 (2016). JNRSDS 0270-6474 10.1523/JNEUROSCI.1099-16.2016 27605612 PMC5013186

[61] Barthas F. Kwan A. C., “Secondary motor cortex: where ‘sensory’ meets ‘motor’ in the rodent frontal cortex,” Trends Neurosci. 40(3), 181–193 (2017). 10.1016/j.tins.2016.11.006 28012708 PMC5339050

[62] Eggermann E. et al., “Cholinergic signals in mouse barrel cortex during active whisker sensing,” Cell Rep. 9(5), 1654–1660 (2014). 10.1016/j.celrep.2014.11.005 25482555

[63] Nelson A. Mooney R., “The basal forebrain and motor cortex provide convergent yet distinct movement-related inputs to the auditory cortex,” Neuron 90(3), 635–648 (2016). NERNET 0896-6273 10.1016/j.neuron.2016.03.031 27112494 PMC4866808

[64] Reimer J. et al., “Pupil fluctuations track rapid changes in adrenergic and cholinergic activity in cortex,” Nat. Commun. 7, 13289 (2016). NCAOBW 2041-1723 10.1038/ncomms13289 27824036 PMC5105162

[65] Howe M. W. Dombeck D. A., “Rapid signalling in distinct dopaminergic axons during locomotion and reward,” Nature 535(7613), 505–510 (2016). 10.1038/nature18942 27398617 PMC4970879

[66] Parker N. F. et al., “Reward and choice encoding in terminals of midbrain dopamine neurons depends on striatal target,” Nat. Neurosci. 19(6), 845–854 (2016). NANEFN 1097-6256 10.1038/nn.4287 27110917 PMC4882228

[67] Stuart G. et al., “Action potential initiation and backpropagation in neurons of the mammalian CNS,” Trends Neurosci. 20(3), 125–131 (1997). 10.1016/S0166-2236(96)10075-8 9061867

[68] Stuart G. J. Sakmann B., “Active propagation of somatic action potentials into neocortical pyramidal cell dendrites,” Nature 367(6458), 69–72 (1994). 10.1038/367069a0 8107777

[69] Kaiser K. M. Zilberter Y. Sakmann B., “Back-propagating action potentials mediate calcium signalling in dendrites of bitufted interneurons in layer 2/3 of rat somatosensory cortex,” J. Physiol. 535(Pt. 1), 17–31 (2001). JPHYA7 0022-3751 10.1111/tjp.2001.535.issue-1 11507155 PMC2278771

[70] Carter A. G. Sabatini B. L., “State-dependent calcium signaling in dendritic spines of striatal medium spiny neurons,” Neuron 44(3), 483–493 (2004). NERNET 0896-6273 10.1016/j.neuron.2004.10.013 15504328

[71] Llinas R. Sugimori M., “Electrophysiological properties of in vitro Purkinje cell dendrites in mammalian cerebellar slices,” J. Physiol. 305, 197–213 (1980). JPHYA7 0022-3751 10.1113/jphysiol.1980.sp013358 7441553 PMC1282967

[72] Svoboda K. et al., “In vivo dendritic calcium dynamics in neocortical pyramidal neurons,” Nature 385(6612), 161–165 (1997). 10.1038/385161a0 8990119

[73] Hill D. N. et al., “Multibranch activity in basal and tuft dendrites during firing of layer 5 cortical neurons in vivo,” Proc. Natl. Acad. Sci. U. S. A. 110(33), 13618–13623 (2013). PNASA6 0027-8424 10.1073/pnas.1312599110 23904480 PMC3746846

[74] Waters J. et al., “Supralinear ${\mathrm{Ca}}^{2+}$ influx into dendritic tufts of layer 2/3 neocortical pyramidal neurons in vitro and in vivo,” J. Neurosci. 23(24), 8558–8567 (2003). JNRSDS 0270-6474 10.1523/JNEUROSCI.23-24-08558.2003 13679425 PMC6740370

[r75] Svoboda K. et al., “Spread of dendritic excitation in layer 2/3 pyramidal neurons in rat barrel cortex in vivo,” Nat. Neurosci. 2(1), 65–73 (1999). NANEFN 1097-6256 10.1038/4569 10195182

[76] Helmchen F. et al., “In vivo dendritic calcium dynamics in deep-layer cortical pyramidal neurons,” Nat. Neurosci. 2(11), 989–996 (1999). NANEFN 1097-6256 10.1038/14788 10526338

[77] Jia H. et al., “Dendritic organization of sensory input to cortical neurons in vivo,” Nature 464(7293), 1307–1312 (2010). 10.1038/nature08947 20428163

[78] Vetter P. Roth A. Hausser M., “Propagation of action potentials in dendrites depends on dendritic morphology,” J. Neurophysiol. 85(2), 926–937 (2001). JONEA4 0022-3077 10.1152/jn.2001.85.2.926 11160523

[79] Larkum M. E. et al., “Synaptic integration in tuft dendrites of layer 5 pyramidal neurons: a new unifying principle,” Science 325(5941), 756–760 (2009). SCIEAS 0036-8075 10.1126/science.1171958 19661433

[80] Schiller J. et al., “NMDA spikes in basal dendrites of cortical pyramidal neurons,” Nature 404(6775), 285–289 (2000). 10.1038/35005094 10749211

[81] Yuste R. et al., “${\mathrm{Ca}}^{2+}$ accumulations in dendrites of neocortical pyramidal neurons: an apical band and evidence for two functional compartments,” Neuron 13(1), 23–43 (1994). NERNET 0896-6273 10.1016/0896-6273(94)90457-X 8043278

[r82] Markram H. Sakmann B., “Calcium transients in dendrites of neocortical neurons evoked by single subthreshold excitatory postsynaptic potentials via low-voltage-activated calcium channels,” Proc. Natl. Acad. Sci. U. S. A. 91(11), 5207–5211 (1994). PNASA6 0027-8424 10.1073/pnas.91.11.5207 8197208 PMC43961

[83] Schiller J. et al., “Calcium action potentials restricted to distal apical dendrites of rat neocortical pyramidal neurons,” J. Physiol. 505(Pt. 3), 605–616 (1997). JPHYA7 0022-3751 10.1111/tjp.1997.505.issue-3 9457639 PMC1160039

[84] Xu N. L. et al., “Nonlinear dendritic integration of sensory and motor input during an active sensing task,” Nature 492(7428), 247–251 (2012). 10.1038/nature11601 23143335

[85] Palmer L. M. et al., “NMDA spikes enhance action potential generation during sensory input,” Nat. Neurosci. 17(3), 383–390 (2014). NANEFN 1097-6256 10.1038/nn.3646 24487231

[86] Cichon J. Gan W. B., “Branch-specific dendritic ${\mathrm{Ca}}^{2+}$ spikes cause persistent synaptic plasticity,” Nature 520(7546), 180–185 (2015). 10.1038/nature14251 25822789 PMC4476301

[87] Kerlin A. et al., “Functional clustering of dendritic activity during decision-making,” biorxiv 440396 (2018). 10.1101/440396 PMC6821494 31663507

[88] Murayama M. et al., “Dendritic encoding of sensory stimuli controlled by deep cortical interneurons,” Nature 457(7233), 1137–1141 (2009). 10.1038/nature07663 19151696

[89] Yuste R. Denk W., “Dendritic spines as basic functional units of neuronal integration,” Nature 375(6533), 682–684 (1995). 10.1038/375682a0 7791901

[90] Noguchi J. et al., “Spine-neck geometry determines NMDA receptor-dependent ${\mathrm{Ca}}^{2+}$ signaling in dendrites,” Neuron 46(4), 609–622 (2005). NERNET 0896-6273 10.1016/j.neuron.2005.03.015 15944129 PMC4151245

[91] Chen X. et al., “Functional mapping of single spines in cortical neurons in vivo,” Nature 475(7357), 501–505 (2011). 10.1038/nature10193 21706031

[92] Schiller J. Schiller Y. Clapham D. E., “NMDA receptors amplify calcium influx into dendritic spines during associative pre- and postsynaptic activation,” Nat. Neurosci. 1(2), 114–118 (1998). 10.1038/363 10195125

[93] Bloodgood B. L. Giessel A. J. Sabatini B. L., “Biphasic synaptic Ca influx arising from compartmentalized electrical signals in dendritic spines,” PLoS Biol. 7(9), e1000190 (2009). 10.1371/journal.pbio.1000190 19753104 PMC2734993

[94] Scholl B. Wilson D. E. Fitzpatrick D., “Local order within global disorder: synaptic architecture of visual space,” Neuron 96(5), 1127–1138.e4 (2017). NERNET 0896-6273 10.1016/j.neuron.2017.10.017 29103806 PMC5868972

[95] Wilson D. E. et al., “Orientation selectivity and the functional clustering of synaptic inputs in primary visual cortex,” Nat. Neurosci. 19(8), 1003–1009 (2016). NANEFN 1097-6256 10.1038/nn.4323 27294510 PMC5240628

[96] Iacaruso M. F. Gasler I. T. Hofer S. B., “Synaptic organization of visual space in primary visual cortex,” Nature 547(7664), 449–452 (2017). 10.1038/nature23019 28700575 PMC5533220

[97] Levy M. Schramm A. E. Kara P., “Strategies for mapping synaptic inputs on dendrites in vivo by combining two-photon microscopy, sharp intracellular recording, and pharmacology,” Front. Neural Circuits 6, 101 (2012). 10.3389/fncir.2012.00101 23248588 PMC3521157

[98] Gambino F. et al., “Sensory-evoked LTP driven by dendritic plateau potentials in vivo,” Nature 515(7525), 116–119 (2014). 10.1038/nature13664 25174710

[99] Grienberger C. Chen X. Konnerth A., “NMDA receptor-dependent multidendrite ${\mathrm{Ca}}^{2+}$ spikes required for hippocampal burst firing in vivo,” Neuron 81(6), 1274–1281 (2014). NERNET 0896-6273 10.1016/j.neuron.2014.01.014 24560703

[100] Larkum M. E. Zhu J. J. Sakmann B., “A new cellular mechanism for coupling inputs arriving at different cortical layers,” Nature 398(6725), 338–341 (1999). 10.1038/18686 10192334

[101] Waters J. Helmchen F., “Boosting of action potential backpropagation by neocortical network activity in vivo,” J. Neurosci. 24(49), 11127–11136 (2004). JNRSDS 0270-6474 10.1523/JNEUROSCI.2933-04.2004 15590929 PMC6730284

[102] Stuart G. J. Hausser M., “Dendritic coincidence detection of EPSPs and action potentials,” Nat. Neurosci. 4(1), 63–71 (2001). NANEFN 1097-6256 10.1038/82910 11135646

[103] Larkum M. E. Kaiser K. M. Sakmann B., “Calcium electrogenesis in distal apical dendrites of layer 5 pyramidal cells at a critical frequency of back-propagating action potentials,” Proc. Natl. Acad. Sci. U. S. A. 96(25), 14600–14604 (1999). PNASA6 0027-8424 10.1073/pnas.96.25.14600 10588751 PMC24482

[104] Chiu C. Q. et al., “Compartmentalization of GABAergic inhibition by dendritic spines,” Science 340(6133), 759–762 (2013). SCIEAS 0036-8075 10.1126/science.1234274 23661763 PMC3752161

[105] Murayama M. Larkum M. E., “Enhanced dendritic activity in awake rats,” Proc. Natl. Acad. Sci. U. S. A. 106(48), 20482–20486 (2009). PNASA6 0027-8424 10.1073/pnas.0910379106 19918085 PMC2777965

[106] Baker C. A. et al., “Cellular resolution circuit mapping with temporal-focused excitation of soma-targeted channelrhodopsin,” Elife 5, e14193 (2016). 10.7554/eLife.14193 27525487 PMC5001837

[107] Broussard G. J. et al., “In vivo measurement of afferent activity with axon-specific calcium imaging,” Nat. Neurosci. 21(9), 1272–1280 (2018). NANEFN 1097-6256 10.1038/s41593-018-0211-4 30127424 PMC6697169

[108] Zipfel W. R. Williams R. M. Webb W. W., “Nonlinear magic: multiphoton microscopy in the biosciences,” Nat. Biotechnol. 21(11), 1369–1377 (2003). NABIF9 1087-0156 10.1038/nbt899 14595365

[109] Sheffield M. E. Dombeck D. A., “Calcium transient prevalence across the dendritic arbour predicts place field properties,” Nature 517(7533), 200–204 (2015). 10.1038/nature13871 25363782 PMC4289090

[110] Lu R. et al., “Video-rate volumetric functional imaging of the brain at synaptic resolution,” Nat. Neurosci. 20(4), 620–628 (2017). 10.1038/nn.4516 28250408 PMC5374000

[111] Dana H. et al., “High-performance calcium sensors for imaging activity in neuronal populations and microcompartments,” Nat. Methods 16, 649–657 (2019).1548-7091 10.1038/s41592-019-0435-6 31209382

